# Using In Silico Molecular Docking to Explain Differences in Receptor Binding Behavior of HHC and THCV Isomers: Revealing New Binding Modes

**DOI:** 10.3390/ph17050637

**Published:** 2024-05-15

**Authors:** Mehdi Haghdoost, Yossef López de los Santos, Megan Brunstetter, Morgan L. Ferretti, Matthew Roberts, Marcel O. Bonn-Miller

**Affiliations:** 1 Nalu Bio Inc., 38 Keyes Avenue, Suite 117, San Francisco, CA 94129, USA; mmhaghdoost@gmail.com (M.H.); matthew@nalubio.com (M.R.); 2 Biological and Environmental Science and Engineering Division, King Abdullah University of Science and Technology (KAUST), Thuwal 23955-6900, Saudi Arabia; yossefls.biotechnologyconsultancy@protonmail.com; 3 Charlotte’s Web, 700 Tech Court, Louisville, CO 80027, USA; megan.brunstetter@charlottesweb.com; 4 Department of Psychological Science, University of Arkansas, 216 MEMH, Fayetteville, AR 72701, USA; ferretti@uark.edu

**Keywords:** tetrahydrocannabivarin, hexahydrocannabinol, CB1 receptor, agonist, antagonist, molecular docking

## Abstract

Even slight structural differences between phytocannabinoid isomers are usually enough to cause a change in their biological properties. In this study, we used in vitro CB1 agonism/antagonism assays to compare the receptor binding functionality of THCV (tetrahydrocannabivarin) and HHC (hexahydrocannabinol) isomers and applied molecular docking to provide an explanation for the difference in the activities. No CB1 agonism was observed for ∆9- and ∆8-THCV. Instead, both isomers antagonized CP 55940, with ∆9-THCV being approximately two times more potent than the ∆8 counterpart (IC_50_ = 52.4 nM and 119.6 nM for ∆9- and ∆8-THCV, respectively). Docking simulations found two binding poses for THCV isomers, one very similar to ∆9-THC and one newly discovered pose involving the occupation of side pocket 1 of the CB1 receptor by the alkyl chain of the ligand. We suggested the latter as a potential antagonist pose. In addition, our results established 9*R*-HHC and 9*S*-HHC among partial agonists of the CB1 receptor. The 9*R*-HHC (EC_50_ = 53.4 nM) isomer was a significantly more potent agonist than 9*S* (EC_50_ = 624.3 nM). ∆9-THC and 9*R*-HHC showed comparable binding poses inside the receptor pocket, whereas 9*S*-HHC adopted a new and different binding posture that can explain its weak agonist activity.

## 1. Introduction

**Figure 1:**
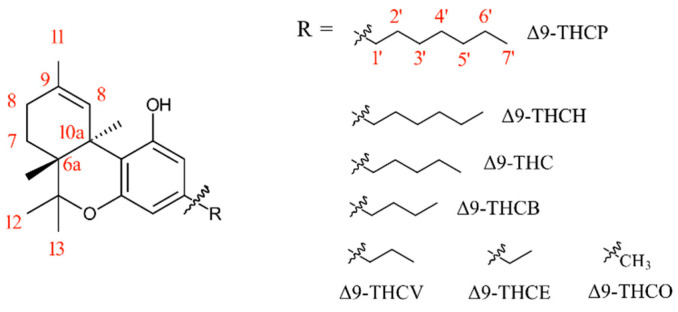
Structure of ∆9 isomers of THC-based molecules with various alkyl chain lengths, including some atom numberings in red.

Many structural factors affect the biological properties of cannabinoids. Early structure–activity studies proved the importance of the alkyl side chain on the CB1 binding affinity of tetrahydrocannabinol (THC) molecules [1]. The findings can be used to explain the difference in psychoactivity of THC, tetrahydrocannabiphorol (THCP), tetrahydrocannabivarin (THCV), and tetrahydrocannabiorcol (THCO; Figure 1); however, most phytocannabinoids possess natural and synthetic isomers with very similar structural backbones. THC, for example, has seven distinguished isomers only depending on the position of the double bond of the cyclohexene ring: ∆10, ∆9, ∆8, ∆7, ∆6a(10a), ∆6a(7), and ∆9(11). To add another layer of complexity, each isomer can have multiple diastereomers and enantiomers by changing chirality at carbons 10a, 9, and 6a (for carbon numbering, see Figure 1). For example, ∆7-THC has three stereogenic carbon atoms, and eight stereoisomers can be present.

Information regarding the impact of minor structural changes, such as the position of double bonds and stereochemistry of chiral centers, on the receptor binding and biological properties of cannabinoids is scarce. In a rare study by Reggio and colleagues, the orientation of the C9 substituent is suggested as the main reason for the difference in cannabinoid activity of some cannabinoids with close structures [2]. Despite this knowledge gap, the literature review suggests that even small changes in the structure can entirely change the biological properties of phytocannabinoids. For instance, we have recently shown that in contrast to ∆9-THC, the ∆10-THC isomer is incapable of agonizing the CB1 cannabinoid receptor and instead acts as a potent antagonist of CB1 [3]. In addition, *K*i for displacing CP 55940 at CB1 by (−)-CBD, the natural enantiomer of CBD, is fivefold higher than the synthetic enantiomer (+)-CBD [4].

**Figure 2:**
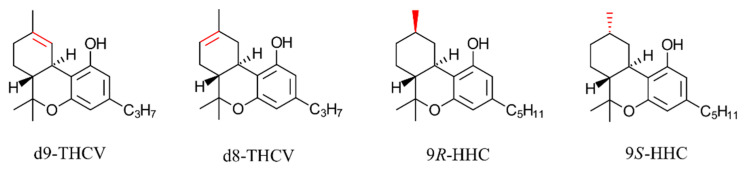
Structure of common THCV and HHC isomers. Structural differences are highlighted in red.

The lack of solid understanding of differences in the biological activity of cannabinoid isomers has become a point of concern recently as various synthetic and natural isomers of phytocannabinoids have entered the North American recreational market. Tetrahydrocannabivarin (THCV) and hexahydrocannabinol (HHC) are two prominent examples [5]. THCV, which has become popular due to its reported capability to reduce blood sugar and appetite in animal models [6,7], possesses two main isomers depending on the position of the double bond: ∆9-THCV and ∆8-THCV (Figure 2). The ∆9-THCV isomer is a phytocannabinoid first discovered in *Cannabis sativa* by Merkus in 1971 [2]. Surprisingly, no report mentions the presence of ∆8-THCV in cannabis plants. Instead, ∆8-THCV is made by simple acid isomerization of CBDV, an approach commonly taken where cannabis cultivation is considered illegal. It has been recently shown that both Δ9-THCV and Δ8-THCV are CB1 receptor antagonists, but with a twofold difference in IC_50_ [8], suggesting an expected difference in biological potency and human effect of isomers. 

Both isomers may prove to have therapeutic utility. However, their difference in potency suggests that they should be separately considered in patient and consumer applications. At a minimum, both health care providers and consumers need to have transparency about which isomers are present in products. Currently, most, if not all, commercial products containing THCV in the North American market fail to mention which isomer(s) of THCV are included. 

HHC follows a similar story. It is a phytocannabinoid [9], but due to the trace quantity in the plant, HHC is typically synthesized by THC hydrogenation. HHC has two isomers depending on the stereochemistry of the newly formed chiral center during the hydrogenation (C9): 9*R*-HHC and 9*S*-HHC (Figure 2). HHC is popular as a psychoactive alternative to THC. Products containing 25–500 mg HHC are present in the US market to provide a similar “high” effect to THC. It is known from early studies of psychoactivity in rhesus monkeys that the 9*R* isomer is significantly more potent than the 9*S* counterpart [10]. This observation has been recently supported by in vitro cAMP assays showing that 9*R*-HHC is a more potent CB1 agonist than the 9*S* counterpart [11]; however, the reason behind any variation in activity between 9*R*-HHC and 9*S*-HHC has yet to be explored. As with THCV, most, if not all, commercial HHC products also do not provide any information about the HHC isomer used in the production stage, although it is clear that the human effect of these products depends strongly on the ratio of isomers. 

To shed more light on the difference in the biological activity of THCV and HHC isomers, we studied their CB1 receptor binding functionality and used in silico molecular docking to explain the obtained data. 

## 2. Results and Discussion

### 2.1. THCV

**Figure 3:**
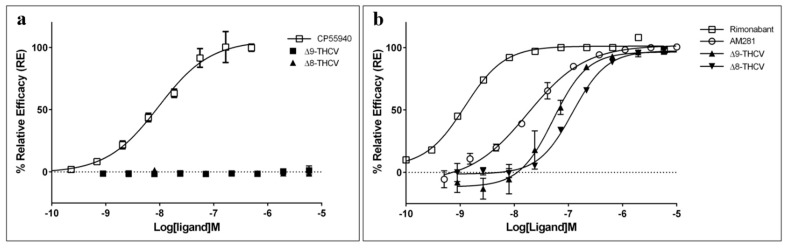
(**a**) Agonist mode CB1 β-arrestin assay for CP 55940 and THCV isomers after 90 min incubation time using PathHunter^®^ assay; 100% relative activity was normalized to the maximum (±)-CP 55,940 stimulation and 0% relative activity to compound vehicle control. (**b**) Antagonist mode CB1 β-arrestin assay for synthetic antagonists (rimonabant and AM-281) and THCV isomers after 30 min incubation time followed by 90 min (±)-CP 55,940 challenge (5.04 nM) using PathHunter^®^ assay; 100% relative activity was normalized to the maximum AM-281, and 0% relative activity was normalized to compound vehicle control. Error bars represent the standard deviation of three independent measurements.

The agonist property of ∆9-THCV and ∆8-THCV was assessed using the β-arrestin recruitment assay in CB1 expressing CHO-K1 cell line, in which both isomers failed to show any significant agonist property up to 5 µM (Figure 3a). An insignificant increase from baseline was observed for ∆9-THCV (E_max_ of 1.19%), suggesting that this isomer may show a weak agonist property at higher concentrations; however, higher concentrations were not tested, as most cannabinoids are known to exert some in vitro cell cytotoxicity at a dose higher than 5 µM [12]. Instead, both isomers were capable of antagonizing the agonist effect of CP 55940, a synthetic cannabinoid and full agonist of CB1, with surprisingly similar maximum relative efficiency to AM-281 and rimonabant, two synthetic antagonists/inverse agonists of human CB1 receptor (Figure 3b, Table 1). The obtained antagonist values for ∆9-THCV and ∆8-THCV fitted greatly (R^2^ > 0.98) into a four-parameter log-logistic model (antagonist concentration) vs. response curve, allowing calculation of IC_50_ and Hill slope values. The ∆9 isomer was found to be approximately two times stronger an antagonist than the ∆8 counterpart, realizing an IC_50_ of 52.4 nM (119.6 nM for ∆8-THCV). This difference in antagonist activity has also been highlighted recently by Walsh and colleagues, reporting that, compared to ∆8-THCV, ∆9-THCV is more potent in antagonizing WIN 55212-2 (another full agonist of CB1) [8]. The ∆9 isomer was, however, a ~3× and ~45× less potent antagonist than AM-281 and rimonabant, respectively.

Another aspect of the data that caught our attention was the antagonist Hill slope value of THCV isomers, which realized values significantly higher than unity (Table 2). These values suggest a complex binding profile for ∆9-THCV and ∆8-THCV, proposing the possibility of more than one binding site [13]; however, considering the limitation of the Hill equation [14], further study is required to confirm this hypothesis.

**Figure 4:**
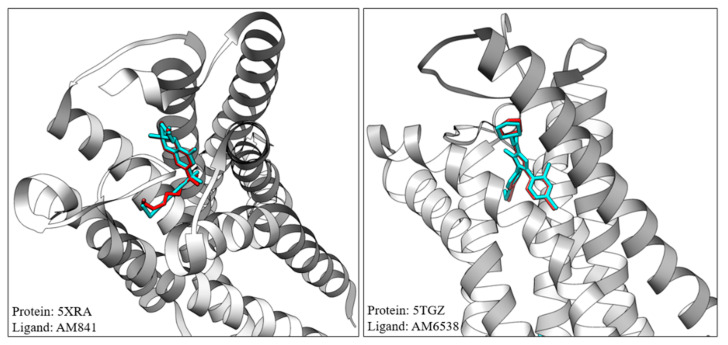
Reproduction of crystalized ligand binding mode using validated molecular docking simulations. Cyan: crystallized ligand extracted from PDB file; red: model prediction of ligand posture. Some residues were omitted for clarity.

Although the IC_50_ for THCV isomers is higher than that of AM-281 and rimonabant, their nanomolar range suggests that both isomers can be considered potent antagonists; however, the CB1 antagonist effect of THCV has always been the subject of debate in the scientific community. In a review of ∆9-THC and ∆9-THCV functionality at cannabinoid receptors, Pertwee concluded that ∆9-THCV can act as a CB1 antagonist at low doses but shows agonist-like CB1 activation at high concentrations [15], an utterly distinguished functionality compared to ∆9-THC. THCV is reported to have a weaker affinity toward CB2 than THC [15]. However, similar to CB1, some studies have reported THCV as a potent agonist of CB2 [16] and some as an antagonist [17]. The dose-dependent dual agonist/antagonist property, which to some extent has been overlooked by other studies, reveals the potential of two binding modes for ∆9-THCV. Raïch and colleagues suggest that the difference in the functionality of THC and THCV at the CB1 receptor originated from variation in binding modes that results in qualitatively different effects depending on the signaling pathway engaged upon receptor activation [18]. We applied in silico molecular docking simulations to shed more light on the difference between the interaction of THCV isomers and THC with the CB1 receptor. The in silico molecular docking method was first optimized and validated using a practical guide outlined by Bender and colleagues [19]. As shown in Figure 4, the model was capable of predicting the binding site and binding posture of crystallized ligands in both agonist and antagonist states with good accuracy, producing ligand root mean square deviation (RMSD, crystal structure against predicted docking pose, calculated with Chimera) of 0.580 and 0.616 Å for agonist (AM841) and antagonist (AM638) ligands, respectively.

**Figure 5:**
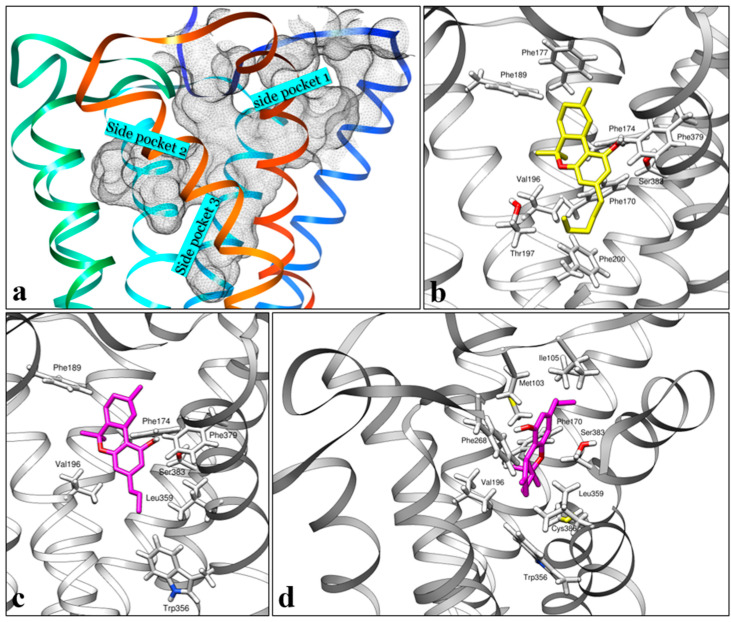
(**a**) Binding pocket analysis of CB1 receptor (agonist state, 5XRA) with side pocket numbering. (**b**) Binding poses of ∆9-THC and (**c**) ∆9-THCV inside the orthosteric ligand binding site of the CB1 receptor in the agonist state (5XRA). (**d**) Binding poses of ∆9-THCV inside the orthosteric ligand binding site of the CB1 receptor in the antagonist state (5TGZ). Some parts of the helices were removed for clarity.

The orthosteric binding pocket analysis of the CB1 receptor using CASTp 3.0 [20] shows a binding core and three main binding side channels (Figure 5a). After validation of our in silico model, we studied the interaction of THCV isomers and THC with the binding pocket of the CB1 receptor in both agonist (5XRA) and antagonist (5TGZ) states. In the agonist mode, ∆9- and ∆8-THCV show very similar poses inside the orthosteric site of the CB1 receptor to that of ∆9-THC (Figure 5b,c). This pose, which involves hydrogen binding between the phenol OH group and Ser383^7.39^, as well as π−π interaction of the benzene ring with phenylalanines, Phe170^2.57^, and Phe379^7.35^ (Figure 5c), has been discussed previously by other research groups [21]. In this binding pose (hereby called agonist pose), the alkyl chain of the cannabinoids occupies the narrow side pocket 3; however, a new energetically favorable pose was discovered when we studied the interaction of THCVs with the antagonist state of the CB1 receptor. In this new pose (hereby called antagonist pose), demonstrating the highest docking score, the alkyl chain is not pointed to the center of the receptor but instead occupies the side pocket 1 (Figure 5d). On the one hand, this led to interaction with new lipophilic groups, in particular, strong interactions with Phe268^ECL2^ (1.993 Å, closest distance between ligand and residue), Met103 (2.475 Å), and Ile105 (2.658 Å). Among these residues, Met103 and Ile105 belong to the N-terminus of the receptor. On the other hand, the THCV is deprived of interacting with Phe379^7.35^, which, according to mutagenesis studies, is essential in demonstrating agonist properties [22]. In addition, occupation of side pocket 1 and interactions with ECL2/N-terminus residues have been previously reported in the CB1 docking simulation of synthetic antagonists from the AM family [23], prompting us to suggest that this pose is responsible for the observed antagonist property of THCV isomers. The presence of two binding modes can be used to explain the dual CB1 agonist/antagonist functionality of ∆9-THCV in some in vitro assays [15]. 

CASTp 3.0 analysis shows a binding pocket area of 1465.3 Å for antagonist-bound CB1 (5TGZ) and 1201 Å for agonist-bound CB1 (5XRA). In the analysis of the crystal structure of CB1 receptors, Hua et al. also noted a 53% increase in the volume of the ligand binding pocket in the antagonist/rest state relative to the agonist-bound state [24]. We believe the antagonist pose discovered in this study is likely possible due to the bigger pocket size of the receptor in the antagonist state. 

**Figure 6:**
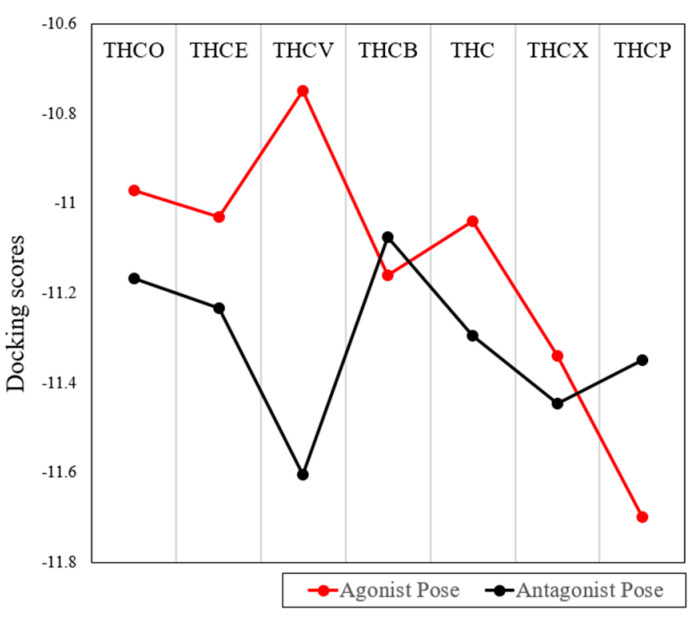
Changes in docking scores of agonist and antagonist pose in a series of THC-based molecules with various alkyl chain lengths. Docking scores are presented in kcal/mol.

Interestingly, when we conducted docking simulations between ∆9-THC-based molecules bearing different alkyl chain lengths (1 to 7, Figure 1) and 5TGZ (CB1 receptor in antagonist state), for all ligands, the antagonist pose also appeared among the top three poses. To investigate further, we compared the docking score of agonist and antagonist poses across this series of cannabinoids (Figure 6). We discovered the docking scores do not follow a linear trend while providing a very good prediction of in vitro experimental data. In the antagonist pose, ∆9-THCV has the best docking score in the series while showing a poor docking score in the agonist mode, consistent with its antagonist properties in vitro. The difference between the binding score of agonist and antagonist poses also suggests that ∆9-THCB (Δ9-tetrahydrocannabutol) and ∆9-THCP should be stronger agonists than ∆9-THC, with ∆9-THCP being the strongest agonist in the series, again in agreement with the experimental data [21,25].

It is noteworthy that the docking score for the antagonist pose of ∆8-THCV (−11.542 kcal/mol) is slightly worse than that of ∆9-THCV (−11.605 kcal/mol), supporting slightly higher IC_50_ of ∆8-THCV in the in vitro antagonist assay. 

### 2.2. HHC

**Figure 7:**
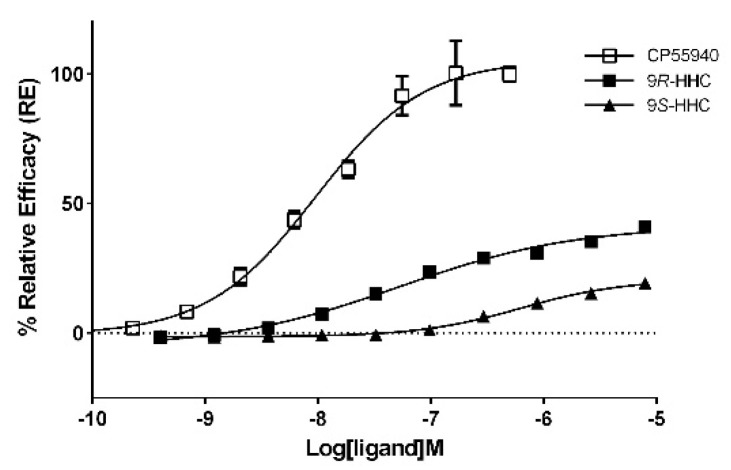
Agonist mode CB1 β-arrestin assay for CP 55940 and HHC isomers after 90 min incubation time using PathHunter^®^ assay; 100% relative activity was normalized to the maximum (±)-CP 55,940 stimulation and 0% relative activity to compound vehicle control. Error bars represent the standard deviation of three independent measurements.

Both 9*R*-HHC and 9*S*-HHC isomers demonstrated agonist activity at the CB1 receptor in the β-arrestin recruitment assay. The obtained antagonist values for 9*R*-HHC and 9*S*-HHC fitted greatly (R^2^ > 0.98) into four-parameter log-logistic model (agonist concentration) vs. response curve, allowing calculation of EC_50_ and Hill slope values (Figure 7). The 9*R* isomer was significantly more potent, showing ~12× lower EC_50_ (53.4 nM) than the 9*S* counterpart (624.30 nM). Relative to the full agonist CP 55940, the *R* isomer also led to higher maximum receptor activation than 9*S*-HHC (41.51% vs. 20.58% for *S*; Table 2), presenting a surprisingly similar CB1 activation profile and potency to ∆9-THC [26]; however, both isomers were significantly less potent than CP 55940 and can be classified as moderate (9*R*) and weak (9*S*) partial agonists of the CB1 cannabinoid receptor. In a recent study, Nasrallah and Garg reported smaller agonist EC_50_ values for 9*R*-HHC (3.4 nM) and 9*S*-HHC (57 nM) using a cAMP assay, but their data also similarly suggest approximately 16-fold higher agonist potency for 9*R*-HHC [11]. 

**Figure 8:**
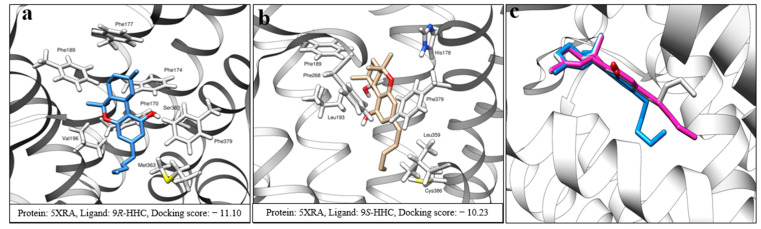
3D binding poses of (**a**) 9*R*-HHC and (**b**) 9*S*-HHC inside the CB1 receptor pocket. (**c**) Three different possible orientations of Δ9-THC alkyl chain inside the CB1 pocket with close docking scores. Some parts of the helices were removed for clarity. Docking scores are presented in kcal/mol.

Differences in EC_50_, maximum RE percentage, and Hill slope values of 9*R*- and 9*S*-HHC encouraged us to study their interaction with the agonist mode of the CB1 receptor (5XRA) using a molecular docking approach. We discovered that 9*R*-HHC and ∆9-THC adopt very similar poses inside the orthosteric ligand binding site of the CB1 receptor (Figure 5b and Figure 8a), explaining their similar in vitro receptor binding properties. In this pose, the aromatic group of the phenolic ring is almost sandwiched between two phenylalanine amino acids, Phe170^2.57^ and Phe379^7.35^ (Figure 8a). Other lipophilic phenylalanine residues (Phe174^2.61^, Phe177^2.64^, Phe189^3.25^, and Phe268^ECL2^), are involved in stabilizing methyl groups C11, C12, and C13 (for atom numbering, see Figure 1). An important polar interaction in this pose is the strong binding of the OH group with the amino acid Ser383^7.39^. The first three carbons of the pentyl chain (C1′, C2, and C3′) have close contact with Val196^3.32^; however, we noticed that for both 9*R*-HHC and ∆9-THC structures, two terminal carbons (C4′ and C5′) could easily rotate to produce slightly different poses with similar docking scores inside the receptor pocket (Figure 8c). A previous docking study by Linciano and coworkers suggested a very similar variation in the position of the aliphatic side chain as the main reason for the difference in CB1 affinity of Δ9-THC and Δ9-THCB [21]. 

Surprisingly, the docking study revealed an entirely different binding pose for 9*S*-HHC (score: −10.23 kcal/mol), in which the ligand seems to be rotated inside the pocket (Figure 8b). This pose also shows a lower docking score than 9*R*-HHC (score −11.10 kcal/mol) and Δ9-THC poses (score: −11.04 kcal/mol), predicting a weaker affinity of 9*S*-HHC. In this unique pose, the aromatic resorcinol ring is again involved in an edge-to-face π−π interaction with Phe379^7.35^. Interestingly, although a somewhat similar array of amino acids is involved in the structure stabilization of 9*R*-HHC and 9*S*-HHC, these amino acids interact with different parts of these molecules. For example, Phe189^3.25^ interacts with C12 and C13 of 9*R*-HHC, but it is in proximity to C11 in the 9*S*-HHC pose. However, the significant difference in the 9*S*-HHC pose is the lack of an H-bond between the phenolic OH of 9*S*-HHC and Ser383^7.39^. In fact, the rotation of the molecule inside the receptor has deprived the OH group from making any meaningful polar interaction with the CB1 receptor. As the previous structure–activity relationship has shown that the phenolic OH group plays a vital role in the affinity of THC-like molecules toward the CB1 receptor, we believe that the lack of polar interaction with Ser383^7.39^ is the main reason behind the weak agonist property of 9*S*-HHC and its low affinity toward the CB1 receptor [27]. In addition, it has been shown that removing the OH group from Δ9-THC does not affect the affinity toward the CB2 receptor. Our molecular docking, as a result, predicts higher CB2 selectivity for 9*S*-HHC than for 9*R*-HHC. This prediction has been recently confirmed by in vitro studies showing 1.7 times CB2 selectivity for 9*S*-HHC and 1.2 for 9*R*-HHC [11].

## 3. Materials and Methods

### 3.1. PathHunter^®^ Agonist and Antagonist Assay

For agonist and antagonist assays, PathHunter^®^ CHO-K1 CNR1 (Eurofins DiscoverX, Fremont, CA, USA, Catalog number: 93-0959C2) β-Arrestin cells were expanded from freezer stocks according to standard procedures provided by Eurofins. The cell line manual was followed for cell growth (including cell culture media and supplementation, the handling and preparation of cells, etc.), test procedure, and signal detection [28]. Cells were seeded in a total volume of 20 µL (5000 cells) into white-walled, 384-well microplates and incubated at 37 °C overnight prior to testing. After preparing stock acetonitrile solution at 1 mg/mL concentration of ligand, compound intermediate concentrations were achieved by a 10-point series of 3-fold compound serial dilutions in a compound dilution buffer in a separate dilution plate. 

For the agonist assay, the concentration of each dilution was prepared at 5X of the final screening concentration. An amount of 5 µL of samples was added to cells (highest final concentration = 5 µM, maximum acetonitrile = 0.2 *v*/*v*%) and incubated at 37 °C for 90 min (5% CO_2_). To generate the assay signal, 12.5 µL of working detection solution was added to the cells, which were then left to incubate for an hour at room temperature in the dark. The signal detection was carried out using a PerkinElmer Envision instrument to measure chemiluminescence.

For antagonist determination, cells were pre-incubated with THCV isomers, followed by agonist challenge at the EC_80_ concentration. Each THCV dilution was prepared at a 10X concentration relative to the intended final concentrations for screening. An amount of 2.5 µL of these samples were introduced into the cells, achieving a maximum final concentration of 5 µM for THCV and ~0.2 *v*/*v*% for acetonitrile. The assay plate was incubated at 37 °C for 30 min in an atmosphere containing 5% CO_2_. An amount of 2.5 µL of CP 55940 stock solution (10× final concentration) was added to the cells to produce a final concentration equal to EC_80_ (5.04 nM, previously calculated using agonist assay). Cells were incubated for 90 min at 37 °C and 5% CO_2_. To generate the assay signal, 12.5 µL of working detection solution was added to the cells, which were then left to incubate for an hour at room temperature in the dark. The signal detection was carried out using a PerkinElmer Envision (Waltham, MA, USA) instrument to measure chemiluminescence.

The pure ∆8-THCV, ∆9-THCV, and HHC isomers were purchased as analytical standards (in acetonitrile) from Cayman Chemicals (Ann Arbor, MI, USA) [29]. CP 55940 and AM281 were provided by Eurofins (Fremont, CA, USA).

### 3.2. CB1 Cannabinoid Receptor Refinement and Molecular Analysis with THCV and HHC Isomers

The cannabinoid CB1 receptor structural templates were obtained from the Protein Data Bank. The antagonist-bound CB1 protein (5TGZ) [23] was used for antagonist state studies. For agonist mode, four agonist-bound CB1 proteins (5XR8 [24], 5XRA [24], 7V3Z [30], and 8GHV [31]) were investigated, and 5XRA was selected because it produced the best results in the validation process. Some protein structures had a low resolution of 2.8 Å and a medium quality according to the vwPDB validation protocol (according to PDB percentile ranks). Parameters such as side chain outliers and clashcores needed to be refined prior to molecular docking analysis to obtain the most reliable results. The Chimera (RBVI, University of California, San Francisco, CA, USA) Dock Prep function was used to prepare receptors for the docking simulations. Solvent and water molecules were removed. The incomplete side chain was replaced with the Dunbrack 2010 rotamer library [32]. Hydrogen atoms were added considering steric factors and H-bonds. Charges were assigned using the AMBER ff99bsc0 force field [33]. For molecular refinement, we applied molecular dynamic simulation (MD) to refine the overall final model. For MD refinement, we applied the YASARA2 force field and the WHAT-IF protein quality check package [34]. The Yasara2 force field has been optimized to increase the atomic accuracy of protein structures based on multidimensional potentials based on the enormous knowledge curated and deposited in the PDB. Once the CB1 cannabinoid receptor protein model was refined, we performed energy minimization as a prerequisite for molecular docking analysis. The minimization process was performed using the same force field used in the refinement stage with the YASARA Structure molecular suite (YASARA Biosciences GmbH, Wien, Austria). Autodock Vina [35] was used for the molecular docking analysis. The performance of three different scoring functions (vina, vinardo [36], and a recently reported custom empirical set [37]) was assessed during the docking method validation, and the scoring function that performed the best in the validation process was selected for docking analysis. For the costume empirical set, the gauss1, gauss2, repulsion, hydrophobic, hydrogen bond, and rotation values were set at −0.049811, −0.007218, 0.756221, −0.031562, −0.469951, and 0.025722, respectively. 

**Figure 9:**
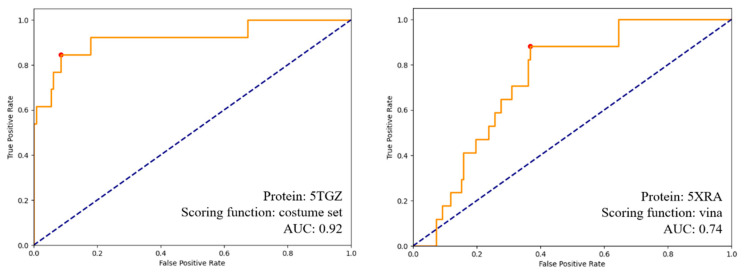
ROC curves for two models used for molecular docking plotting the rate of true positives found against decoys found. The orange line shows model performance, while the dashed blue line represents the random classifier.

For validation of the docking method, molecular docking was first conducted on a series of agonist/antagonist active and decoy ligands (250–300 ligands, active-to-decoy ratio of 1:10), and area under the curves (AUCs) in receiving operating characteristic (ROC) graphs were used to analyze the success of each model and scoring function [19]. Decoy ligands were obtained from the DUD.E database [38]. AUC data and ROC graphs are presented in Table 3 and Figure 9, respectively. To prepare for docking simulations, the 3D structure of ligands was built from SMILES strings using Open Babel 3.1.1 [39], and Gasteiger charges were assigned to each molecule.

Before performing the in silico study, the ligand molecules, as well as the template structure, were prepared. During this phase, parameters such as protonation state, length and angle of atomic bonds were corrected, and flexible atomic bonds were assigned to allow conformational sampling of the ligand molecule chains during molecular docking analysis. A cavity prediction algorithm was used to identify the protein cavity as the binding target in the CB1 protein receptor [40]. Grid box size of 30 × 30 × 30 and exhaustiveness of 50 were used for all molecular docking calculations. No meaningful improvement in docking scores was observed using larger exhaustiveness values. The most favorable (energetically) and best-scoring poses were selected, analyzed, and compared between the different ligands tested. 

## 4. Conclusions

In conclusion, a minimal structural modification can significantly change the CB1 receptor binding behavior of cannabinoids. A slight shift in the position of the double bond that converts ∆9-THCV to ∆8-THCV also reduces the CB1 antagonist potency by twofold. The only structural difference between 9*R*-HHC and 9*S*-HHC is the Chirality of one carbon atom; however, this difference is significant enough to make the 9*R*-HHC isomer a ~10× more potent CB1 agonist than 9*S*. We showed that these variations in activity are directly related to the binding pose ligands taken inside the binding site of the CB1 receptor. In this study, we introduce two new possible binding poses for THCV isomers and 9*S*-HHC, which can explain their unusual CB1 functionality. A lack of strong polar interaction was suggested as the main reason for the drop in CB1 potency of 9*S*-HHC. We also proposed a new antagonist binding pose for THC-based molecules. As the market of minor cannabinoids (cannabinoids that are found in small quantities in *Cannabis sativa*) is rapidly expanding, this study highlights the importance of studying structure–activity relations in the cannabinoid field. To broaden our understanding of the isomeric differences and their pharmacological implications, future studies could explore other cannabinoid receptors, such as CB2, as well as non-cannabinoid receptors that cannabinoids interact with (e.g., serotonin, dopamine, and opioid receptors). Considering that the activity of CYP enzymes can convert cannabinoids to other biologically active compounds, the differences in receptor binding of THCV and HHC metabolites (particularly 11-OH and 11-COOH metabolites) should also be investigated in future studies. 

## Figures and Tables

**Table 1:** IC_50_ values for antagonist effects of THCV isomers, AM281, and rimonabant at CB1 receptor.

	IC_50_ (nM)	Hill Slope	Maximum RE (%)	R Square
∆9-THCV	52.42 (1.11)	1.38 (0.18)	96.46 (2.20)	0.9805
∆8-THCV	119.60 (1.05)	1.37 (0.09)	97.40 (1.16)	0.9950
AM-281	17.71 (1.12)	0.81 (0.07)	101.90 (1.63)	0.9913
Rimonabant	1.22 (1.08)	1.19 (0.09)	101.20 (0.77)	0.9929

Note: Values in parentheses are standard deviations of three independent measurements.

**Table 2:** EC_50_ values for agonist effects of HHC isomers and CP 55940 at CB1 receptor.

	EC_50_ (nM)	Hill Slope	Maximum RE (%)	R Square
9*R*-HHC	53.45 (1.19)	0.56 (0.07)	41.51 (1.70)	0.9887
9*S*-HHC	624.30 (1.12)	0.95 (0.08)	20.58 (0.80)	0.9926
CP 55940	9.48 (1.13)	0.90 (0.10)	105.50 (3.34)	0.9862

Note: Values in parentheses are standard deviations of three independent measurements.

**Table 3:** The area under curve (AUC) values obtained during the docking method validation process. The best values for agonist and antagonist modes are highlighted in green.

	Scoring Function		
PDB Structure	Vina	Vinardo	Costume Set
5TGZ (antagonist bound)	0.87	0.68	0.92
8GHV (agonist bound)	0.60	0.71	0.54
5XRA (agonist bound)	0.74	0.72	0.65
5XR8 (agonist bound)	0.66	0.73	0.59
7Z3V (agonist bound)	0.70	0.65	0.61

## Data Availability

Data are contained within the article.
